# Alternative evolutionary outcomes following endosymbiont‐mediated selection on male mating preference alleles

**DOI:** 10.1111/jeb.13602

**Published:** 2020-02-21

**Authors:** Antje Hundertmark, Sara L. Goodacre, John F. Y. Brookfield

**Affiliations:** ^1^ School of Life Sciences University of Nottingham, University Park Nottingham UK

**Keywords:** cytoplasmic incompatibility, evolutionary model, male preference, *Wolbachia*

## Abstract

In many arthropods, intracellular bacteria, such as those of the genus *Wolbachia*, may spread through host populations as a result of cytoplasmic incompatibility (CI). Here, there is sterility or reduced fertility in crosses between infected males and uninfected females. As the bacterium is maternally inherited, the reduced fertility of uninfected females increases the frequency of the infection. If the transmission fidelity of the bacterium is less than 100%, the bacterium cannot invade from a low frequency, but if its frequency exceeds a threshold, it increases to a high, stable, equilibrium frequency. We explore the expected evolutionary dynamics of mutant alleles that cause their male bearers to avoid mating with uninfected females. For alleles which create this avoidance behaviour conditional upon the male being infected, there is a wide zone of parameter space that allows the preference allele to drive *Wolbachia* from the population when it would otherwise stably persist. There is also a wide zone of parameter space that allows a joint stable equilibrium for the *Wolbachia* and a polymorphism for the preference allele. When the male's avoidance of uninfected females is unconditional, the preference allele's effect on *Wolbachia* frequency is reduced, but there is a narrow range of values for the transmission rate and CI fertility that allow an unconditional preference allele to drive *Wolbachia* from the population, in a process driven by positive linkage disequilibrium between *Wolbachia* and the preference allele. The possibility of the evolution of preference could hamper attempts to manipulate wild populations through *Wolbachia* introductions.

## Graphical abstract

An allele causing a male preference for *Wolbachia*‐infected females spreads and can cause an otherwise stable *Wolbachia* infection to be eliminated.

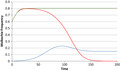

## 1 INTRODUCTION

Many aspects of the biology of arthropods are influenced by the possession of various genera of symbiotic bacteria (Engelstädter & Hurst, 2009; Werren, Baldo, & Clark, 2008). The inheritance of bacteria through the female line has resulted in the spread of bacteria which change the organism's biology in ways that result in a higher level of reproduction through infected females than through uninfected females. Examples are male‐killing and feminization (Engelstädter & Hurst, 2009). In male‐killing, male offspring bearing the endosymbiont die, thus, in some circumstances, either relieving the competition experienced by their sisters or acting as a food source for these sisters. Another phenomenon is cytoplasmic incompatibility (CI), in which matings between infected males and uninfected females result in sterility or low fitness offspring, thus yielding, on average, a higher fertility for females with the endosymbiont, as was seen, for example, in the spread of the bacterium *Wolbachia* through Californian populations of *Drosophila simulans* (Turelli & Hoffmann, 1991). Early theoretical work (Caspari & Watson, 1959; Hoffmann, Hercus, & Dagher, 1998; Turelli & Hoffmann, 1991, 1995) demonstrated that the invasion of a population through a CI‐generating bacterium would face difficulties if the fidelity of maternal transmission was less than 100% or if there is any fitness loss associated with infection. These factors, which tend to reduce infection frequency, are themselves frequency‐independent. However, the advantage that infected females gain through CI increases with the frequency of the infection. This creates a situation where the absence of the bacterium is a stable equilibrium, but there is also potentially a high‐frequency stable equilibrium for infection rate, which the population will move towards provided the initial frequency of infection exceeds an unstable threshold point.

These complex dynamics have been of relevance to the use of *Wolbachia* in reducing insect‐borne disease. It was demonstrated that *Wolbachia*, introduced into *Aedes aegypti*, reduced the ability of the mosquito to transmit dengue fever (Moreira et al., 2009). This result led to the manipulation of wild populations of the mosquito in Queensland, Australia, through the release of very large numbers of *Ae. egypti* infected with the *wMel* strain of *Wolbachia*, which blocks dengue transmission. The numbers had to be high since the *Wolbachia* was not only transmitted with less than 100% frequency, but also imposed a fitness cost on its bearers. Indeed, it was estimated that the unstable equilibrium that had to be exceeded was a *Wolbachia* frequency of around 30%, which was surpassed by the introductions, leading to near‐fixation of the *Wolbachia* in these populations (Hoffmann et al., 2011). Subsequently, there has been evidence that *Wolbachia* can block transmission of Zika viruses in *Ae. aegypti* (Dutra et al., 2016) and, in some host species, some strains of *Plasmodium* (Moreira et al., 2009).

But the persistence of *Wolbachia*, with its harmful effects on host fitness, relies on the host failing to evolve to prevent the bacterium's effects. As with male‐killing and feminization, with cytoplasmic incompatibility, there will be a selective advantage to alleles at nuclear (although not at mitochondrial or W chromosomal) loci that prevent the phenomenon. In addition to there being an advantage for alleles that prevent cytoplasmic incompatibility from occurring in crosses between infected males and uninfected females, it is clear that mutant alleles that will reduce the proportion of these CI‐generating crosses will have an advantage. Champion de Crespigny, Butlin, and Wedell (2005) explored the expected outcomes in a model where a mutation causes females to avoid mating with infected males. The conclusions of this work were that, in the case where there was 100% fidelity in maternal transmission of the bacterium and no fitness costs associated with the infection, the infection would always spread and the mating preference would also spread. When there is less than 100% transmission fidelity, or when there is a fitness cost, the preference may prevent the infection's spread, given initial infection frequencies that would otherwise have permitted this, particularly when the initial frequency of the preference allele is high. The preference allele was always beneficial or neutral in the case where infection was either absent or at 100%. For this reason, there was no stable intermediate equilibrium for the preference allele. It moves to fixation or to a neutral intermediate equilibrium. This is because it was assumed that there was no male limitation, and females with a preference for uninfected males could always find these in a cost‐free way.

Here, we examine a model where there is a preference allele expressed in males for infected females. However, our model for male preference is one that indirectly can impose a cost for the preference. And the male preference will, through its reduction in the proportion of CI matings that uninfected females undergo, reduce the frequency of the *Wolbachia*. (While the model is expressed in terms of *Wolbachia*, it is equally relevant to any other CI‐inducing maternally inherited symbiont.) Males who choose infected females do so by reducing their matings with uninfected females by a proportion *x* relative to their proportions in random mating, and then, to this degree, compete with other males for matings with infected females. The consequence is that, since competition is now higher for access to the infected females, since these are chosen by males with the preference gene, males showing preference will have a reduced chance of mating overall. For this reason, given that the population contains both infected and uninfected females, the preference gene would be harmful when CI is not operating.

The preference shown by males for infected females could be either conditional (i.e. only shown by infected males) or unconditional (shown by all males). The advantage for the preference allele will be greater in the conditional case, but this requires the possibly biologically implausible assumption that the male's behaviour is conditional upon its own infection status. When CI is complete, that is when all offspring of crosses between uninfected females and infected males die, a conditional preference can never be harmful, since the crosses that the preference gene prevents would all have been sterile. If, however, the sterility in CI crosses is not complete, the cost of competing for infected females could outweigh the reduced fitness of CI crosses for males exhibiting a preference. For an unconditional preference, non‐CI‐inducing uninfected with uninfected crosses will also be avoided, and the advantage of the preference will thus be reduced. This creates a subtle but important difference from earlier models (Champion de Crespigny et al., 2005) where preference is always neutral or beneficial.

## 2 METHODS

### 2.1 Modelling

The population dynamics of CI‐inducing *Wolbachia* are complex. A simple analytical model predicts three equilibria for the infection frequency in the absence of mating preference but with less than 100% maternal transmission fidelity, which is shown in the Results section and Appendix 1.

In our model, we combine this CI model with the potential presence of an autosomal allele, *M*, that creates a preference in males for females that are infected. In the conditional model, the male preference only shows itself in males with the *Wolbachia* infection as well as the preference allele. In the unconditional model, the mate preference is shown by uninfected as well as infected males. We thus assume that the single population consists of six genotypes and is panmictic except for any mating preferences shown by males. The genotypes are defined by *U* and *I*, denoting uninfected and infected, and *MM*, *Mm* and *mm* for the genotypes at the preference locus.

The frequencies of the six possible genotypes are the same in males and females, and are *p_UMM_*, *p_UMm_*, *p_Umm_*, *p_IMM_*, *p_IMm_* and *p_Imm_*. The values of these frequencies in the zygotes are changed by loss of *Wolbachia* in transmission to the offspring (which can convert *Wolbachia*‐positive zygotes (i.e., zygotes from *Wolbachia*‐positive mothers) to *Wolbachia*‐negative offspring). *p_U_* and *p_I_* are the proportions of offspring that are uninfected and infected, respectively. *x* represents the strength of male avoidance of uninfected females, and *f* is the fertility of crosses between infected males and uninfected females, where a low *f* indicates strong CI. *c* is the level of inheritance of the *Wolbachia* from infected mothers to offspring and *d* the dominance of the preference allele *M*. *d* is in the range from 0 to 1 and thus allows intermediate dominance as well as full dominance and recessivity.

### 2.2 For a conditional preference

Males with the *IMM* and *IMm* genotypes avoid matings with uninfected females, with avoidance of *x* and *dx*, respectively, and so the proportion of the males competing for matings with uninfected females is 1 − *x*(*p_IMM_* + *dp_IMm_*), which we represent by *C_U_*. Thus, for an uninfected female genotype *i*, of frequency *p_Ui_*, the relative probabilities of mating with different genotypes of males are as follows:

Infected *MM* probability is $\frac{\left(1-x\right)p_{\mathrm{IMM}}}{C_{U}}$
Infected *Mm* probability is $\frac{\left(1-dx\right)p_{\mathrm{IMm}}}{C_{U}}$
Infected *mm* probability is $\frac{p_{\mathrm{Imm}}}{C_{U}}$
Uninfected *MM* probability is $\frac{p_{\mathrm{UMM}}}{C_{U}}$
Uninfected *Mm* probability is $\frac{p_{\mathrm{UMm}}}{C_{U}}$
Uninfected *mm* probability is $\frac{p_{\mathrm{Umm}}}{C_{U}}$

The avoidance, of strength *x* and *dx*, respectively, by infected *MM* and *Mm* males, of uninfected females, will release *MM* and *Mm* males to compete for the infected females. The impact on competition for the infected females of these extra infected males released will be proportional to the relative proportions of uninfected and infected females, represented by *p_U_*/*p_I_*. The competition for infected females, which we call *C_I_*, is thus 1 + (*p_IMM_* + *dp_IMm_*)*xp_U_*/*p_I_*. Thus, for an infected female genotype *i*, the relative probabilities of mating with different genotypes of males are as follows:

For infected *MM* males: $\frac{p_{\mathrm{IMM}}\left(1+xp_{U}/p_{I}\right)}{C_{I}}$
Infected *Mm*s: $\frac{p_{\mathrm{IMm}}\left(1+dxp_{U}/p_{I}\right)}{C_{I}}$
Infected *mm*s: $\frac{p_{\mathrm{Imm}}}{C_{I}}$
Uninfected *MM*s: $\frac{p_{\mathrm{UMM}}}{C_{I}}$
Uninfected *Mm*s: $\frac{p_{\mathrm{UMm}}}{C_{I}}$
Uninfected *mm*s: $\frac{p_{\mathrm{Umm}}}{C_{I}}$

Fertility is *f* in the CI crosses (*I* father and *U* mother). A proportion *c* of the offspring of infected mothers are infected, and a proportion (1 − *c*) are not infected. The proportions of *MM*, *Mm* and *mm* in the offspring are calculated from Mendelian segregation of alleles in their parents.

To test the impact of a finite population size on this model, the program was modified to include a multinomial sampling of genotypes in a finite population of size *N*. The proportions of the six genotypes above are calculated analytically, and then, a population for the next generation is created by multinomially sampling these six genotypes *N* times, with replacement. Then, the numbers in the sample are converted to frequencies that are used for the next generation.

### 2.3 For an unconditional preference

Now all males, whether or not they are infected, avoid mating with the uninfected females. So the competition for uninfected females, *C_U_*, is 1 − *x*(*p_IMM_* + *dp_IMm_* + *p_UMM_* + *dp_UMm_*). Thus, for an uninfected female genotype *i*, of frequency *p_Ui_*, the relative probabilities of mating with different males are as follows:

Infected *MM* probability is $\frac{\left(1-x\right)p_{\mathrm{IMM}}}{C_{U}}$
Infected *Mm* probability is $\frac{\left(1-dx\right)p_{\mathrm{IMm}}}{C_{U}}$
Infected *mm* probability is $\frac{p_{\mathrm{Imm}}}{C_{U}}$
Uninfected *MM* probability is $\frac{\left(1-x\right)p_{\mathrm{UMM}}}{C_{U}}$
Uninfected *Mm* probability is $\frac{\left(1-dx\right)p_{\mathrm{UMm}}}{C_{U}}$
Uninfected *mm* probability is $\frac{p_{\mathrm{Umm}}}{C_{U}}$

The avoidance, of strength *x* or *dx*, by all *MM* and *Mm* males (whether infected or not), of uninfected females, will release *MM* and *Mm* males to compete for the infected females. The competition for infected females, or *C_I_*, is thus 1 + (*p_IMM_* + *dp_IMm_* + *p_UMM_* + *dp_UMm_*)*xp_U_*/*p_I_.* Thus, for an infected female genotype *I*, the relative probabilities of mating with different genotypes of males are as follows:

For infected *MM* males: $\frac{p_{\mathrm{IMM}}\left(1+xp_{U}/p_{I}\right)}{C_{I}}$
Infected *Mm*s:$\frac{p_{\mathrm{IMm}}\left(1+dxp_{U}/p_{I}\right)}{C_{I}}$
Infected *mm*s: $\frac{p_{\mathrm{Imm}}}{C_{I}}$
Uninfected *MM*s: $\frac{p_{\mathrm{UMM}}\left(1+xp_{U}/p_{I}\right)}{C_{I}}$
Uninfected *Mm*s: $\frac{p_{\mathrm{UMm}}\left(1+dxp_{U}/p_{I}\right)}{C_{I}}$
Uninfected *mm*s: $\frac{p_{\mathrm{Umm}}}{C_{I}}$

The model is expressed as a C++ program (see Data S1), into which is input:

The transmission rate, *c*, of the *Wolbachia* from mothers to offspring;
The fertility, *f*, of crosses between infected males and uninfected females;
The initial frequency of the *Wolbachia* infection, *W;*
The initial frequency of the preference mutation, *M;*
The strength of the effects of the preference mutation, *x;*
The dominance, *d*, of *M*, where 1 is fully dominant, and 0 is fully recessive; and
The number of generations of simulation.

From these inputs, the program creates the initial distribution of *p_UMM_*, *p_UMm_*, *p_Umm_*, *p_IMM_*, *p_IMm_* and *p_Imm_* by assuming Hardy–Weinberg equilibrium and linkage equilibrium (although the population that evolves does not show these properties).

### 2.4 Approximate analytical results for the model

It is possible to get analytical results for the model by making assumptions of linkage equilibrium and Hardy–Weinberg proportions. Now, the population can be represented by two variables, *p* and *r*, where *p* represents the proportion of infected animals, and *r* represents the frequency of individuals showing the phenotype of the *M* allele (for the dominant *M* model, if *M* is recessive, *r* represents the frequency of *MM* homozygotes).

## 3 RESULTS

The results presented here include an overview of the established theory of the dynamics of endosymbionts creating cytoplasmic incompatibility. This is followed by the results of simulations of the outcome of the conditional model, and a demonstration of the conditions under which a preference allele could either eliminate or come into a stable equilibrium with a *Wolbachia* infection. Fluctuations around the stable equilibria as a result of finite population size are investigated. Then, unconditional model simulations are examined, showing the far more restricted area of parameter space that allows an unconditional preference allele to eliminate the *Wolbachia* infection. Finally, an approximate analytical model (where linkage equilibrium is assumed) is studied.

### 3.1 Cytoplasmic incompatibility

The fundamental model of CI has been explored by previous authors (Caspari & Watson, 1959; Turelli & Hoffmann, 1995). Our simplified model includes random mating, but the absence of any cost or benefit from the bacterium other than from CI. *p* is the proportion of surviving offspring that come from mothers with *Wolbachia* (called *I* as opposed to *U*). Appendix 1 shows that this system has three equilibrium points, a stable equilibrium at *p* = 0, a high stable equilibrium *p*, and an intermediate unstable equilibrium *p*.

**Figure 1:**
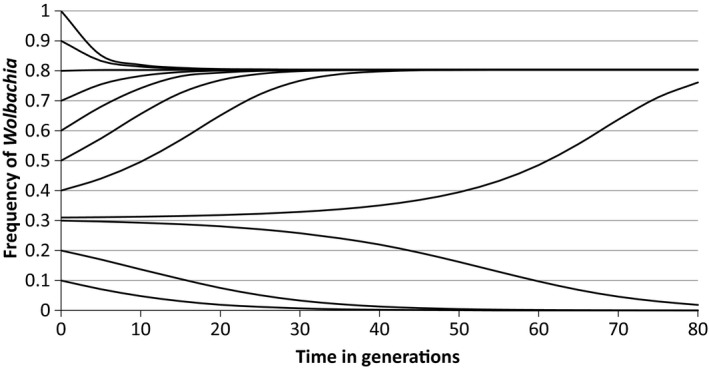
The situations when the transmission rate, *c* = 0.9, and the fertility in cytoplasmically incompatible crosses, *f* = 0.5. There are equilibrium points at 0 (stable), 0.307 (unstable) and 0.804 (stable), which are revealed by changes in the frequency of the *Wolbachia* with time, given different starting frequencies. Thus, for a given set of parameters of the *Wolbachia* infection, the initial frequency will determine whether the population moves to the high stable equilibrium point or whether the bacterium will be lost

An example of these equilibria is shown in Figure 1, based on the model's simulation when the preference allele is absent.

### 3.2 Simulation results: conditional model

In the conditional model, a mutation, *M*, arises that causes males that are *I* to avoid any mating with females that are *U*. We ask whether such a mutation can spread and its impact on the frequency of *Wolbachia.* In each of two sets of conditions that have been considered (i.e. *c* = 0.9, *f* = 0.5; *c* = 0.8, *f* = 0), the outcome observed is that a dominant preference allele (*d* = 1) with full penetrance (*x* = 1) will spread through the population and cause the elimination of the *Wolbachia* from the population.

**Figure 2:**
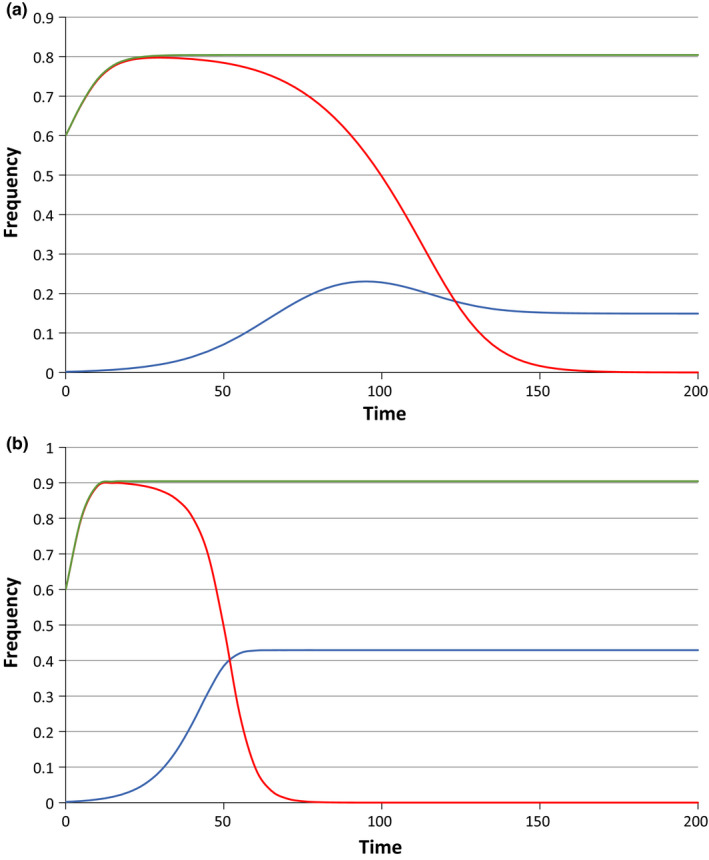
The impact of introduction of a dominant preference allele *M* (of maximum strength: *x* = 1) at a frequency of 0.002 into a population with a *Wolbachia* infection initially at a frequency of 0.6. In (a), the parameters are *c* = 0.9, *f* = 0.5, and in (b), they are *c* = 0.8, *f* = 0. In each figure, the uppermost (green) line shows the movement of the *Wolbachia* frequency to a stable equilibrium in the absence of the preference allele. The red line, showing very similar initial frequency changes, followed by a decline, is the *Wolbachia* frequency when the preference allele is introduced. The lowest (blue) line is the frequency of the preference allele

Figure 2 shows the elimination of the *Wolbachia* infection through the introduction of a dominant preference allele with complete effect (*x* = 1) for the cases of *c* = 0.9, *f* = 0.5, and *c* = 0.8, *f* = 0.0. In Figure 2a, when *f* = 0.5, note that when the *Wolbachia* becomes rare in the population, the preference allele also starts to decline initially. This is because the allele is preventing *I* males from mating with *U* females, causing them to compete for the increasingly scarce *I* females. As *I* females become rarer, the lowered probability of males obtaining a mating starts to outweigh the fitness cost that comes from CI (since, in this case, the CI crosses still have a fertility that is half that of the other crosses). The preference allele also now spends more time in uninfected males, and its effects are thereby diminished. In Figure 2b, where the CI crosses are completely sterile, competing for the scarce *I* females can never be worse than mating with *U* females, and the preference allele can never be disadvantageous. In both cases, once *Wolbachia* has gone, the preference allele is in a neutral equilibrium, since this allele expresses a phenotype only in infected males.

**Figure 3:**
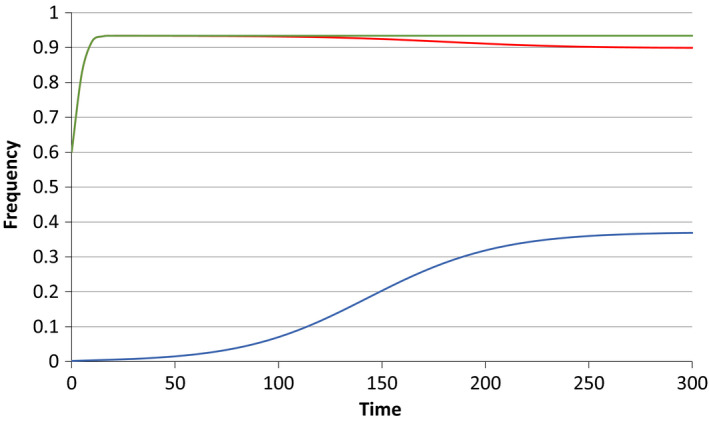
A simulation when *c* = 0.95, *f* = 0.5. The initial frequencies are 0.6 for *Wolbachia* and 0.002 for the preference allele *M*. The upper (green) line shows the rise in *Wolbachia* to its equilibrium frequency of 0.934 when it is introduced in the absence of the preference allele. But with the preference allele (blue) introduced at a frequency 0.002, the *Wolbachia* frequency (red) is reduced to a new equilibrium at 0.898, while the preference allele rises to a stable equilibrium at 0.371

But the elimination of *Wolbachia* is not inevitable under all conditions. If the value for *c* is raised, with *f* still equals to 0.5, there can be a joint stable equilibrium generated, and example of which is shown in Figure 3.

Figure 3 gives an example where both the *Wolbachia* and the preference allele reach a joint stable equilibrium. Here, the preference allele is introduced at a low frequency, but, as we have seen that there are three equilibria that exist for the *Wolbachia* without the preference allele, a stable equilibrium at zero, an unstable equilibrium and a high stable equilibrium, we can ask whether the initial frequency of the preference allele influences how high the initial frequency of the *Wolbachia* has to be in order not to be lost.

**Figure 4:**
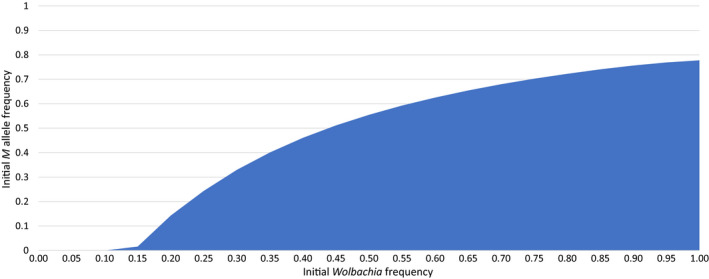
It shows (in blue) the initial frequencies of *Wolbachia* and the preference allele *M* that result in the evolution of the population towards the point of joint stable equilibrium, which is at 0.898:0.371 for *Wolbachia* and *M*, respectively. All populations starting in the white area will evolve to lose the *Wolbachia*

For the case of *c* = 0.95 and *f* = 0.5, where the stable equilibrium point is 0.898:0.371, Figure 4 shows how the initial frequencies of *Wolbachia* and the preference mutation *M* determine whether the population will evolve to the joint stable equilibrium point or whether the *Wolbachia* will be lost. The higher the initial frequency of the preference allele is, the higher the initial frequency of *Wolbachia* has to be in order for the population to evolve to the equilibrium point where the *Wolbachia* persists. In the absence of the preference mutation, the unstable equilibrium point for the Wolbachia frequency is (from 2 in Appendix 1) 0.1186.

Thus, if the population includes *Wolbachia* and a fully penetrant (*x* = 1) preference allele, the two evolutionary outcomes possible are the loss of *Wolbachia*—this leaves an allele frequency of the preference allele that is neutral (as, without *Wolbachia*, the preference allele has no phenotype)—and the persistence of *Wolbachia* and the preference allele in a joint stable equilibrium.

That only two outcomes are possible depends on the effect of the preference mutation, *x*, being 100%. If *x* is less than 1.00, a third outcome is possible, where *Wolbachia* moves to a stable equilibrium, and the preference allele can be fixed in the population. Looking at the *c* = 0.95; *f* = 0.5 model, with diminishing values of *x*, it is seen that, as *x* reduces, the rate of spread of *M* is reduced, and the equilibrium frequency of *M* increases, although the equilibrium value of the *Wolbachia* frequency *p* is unchanged. But as *x* reduces, it reaches a value where *M* will go to fixation. With these low values of *x* causing fixation, the effect of the male preference is attenuated and the *Wolbachia* rises to a higher equilibrium frequency. For the *c* = 0.8; *f* = 0 model, lowering *x* has the effect, apart from slowing the spread of *M*, of allowing *M* to reach a higher frequency before the *Wolbachia* are eliminated. With very low values of *x*, *M* reaches fixation without being able to eliminate the *Wolbachia*.

**Figure 5:**
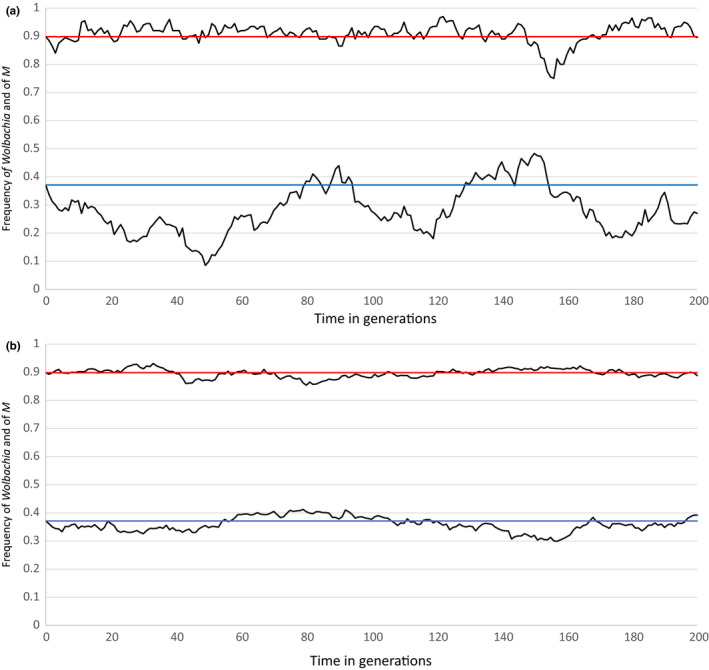
The effect of genetic drift on the situation where *c* = 0.95, *f* = 0.5, d = 1 and *x* = 1. Infinite population size simulations reveal that the joint stable equilibrium has the frequency of *Wolbachia* (in red) at 0.898 and that of *M* (blue) at 0.371. Each figure shows one realization of two hundred generations of genetic drift starting at these equilibrium frequencies with the black lines showing the fluctuations of the frequencies around their equilibrium points. 5a shows an effective size of 200 and (b) an effective size of 2000

All the above results are based on an infinite population size model. Figure 5a,b give examples where the model with *f* = 0.5, *x* = 1, *d* = 1 and *c* = 0.95 was studied in the context of effective population sizes of *N* = 200 and *N* = 2,000, respectively. Changes in the frequency of *Wolbachia* and of the preference mutation *M* are shown for 200 generations, starting at the joint stable equilibrium at 0.898:0.371. As expected, fluctuations around the stable equilibria are greater when the population size is small. Extensive simulations using these parameter values and different population sizes have revealed that *Wolbachia* and *M* are rarely lost when population sizes are 200 or greater. Simulations with population sizes of 150 reveal cases of loss of *M* and of *Wolbachia*. Losses of *Wolbachia* can sometimes be triggered by the frequency of *M* drifting to considerably higher than its equilibrium frequency, which can be followed by a rapid decline and loss of the *Wolbachia*. Figure 5a shows, at around generations 150–170, a decline in the *Wolbachia* frequency following a high frequency of *M* being reached, although in this case the *Wolbachia* recover. If *Wolbachia* is lost, *M* becomes neutral and rapidly drifts to fixation or loss. As can be seen in Figure 5a, *M* fluctuates greatly when population size is small, and is usually seen below its stable equilibrium point. If it is lost, the probability of subsequent loss of *Wolbachia* is greatly diminished. Figure 5b demonstrates that the equilibrium points for *Wolbachia* and *M* are stable, as fluctuations around these points are small and followed by a return to the equilibria.

A key question is thus to investigate the range of parameter space where a preference allele *M*, introduced at low frequency, can cause the elimination of *Wolbachia* from the population in situations (as with *c* = 0.9:*f* = 0.5 and *c* = 0.8:*f* = 0.0) when *Wolbachia* could otherwise persist. Considering cases where the preference allele is dominant (and these results are only very slightly affected by the dominance of the preference allele) and has a complete penetrance (*x* = 1), it is possible to identify values of *c* and *f* that allow the *Wolbachia* to stably persist even when the preference mutation is present.

**Figure 6:**
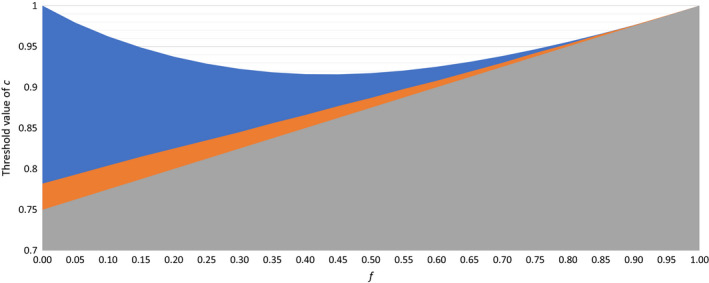
The values of *f* and *c* that make it possible for the *Wolbachia* to persist stably. In the absence of *M*, any *f* and *c* values that fall above the grey zone will allow the persistence of the *Wolbachia*. The upper limit of the grey zone represents *c *= (*f* + 3)/4. The white zone represents *f* and *c* values that allow persistence of *Wolbachia* in the presence of *M* in the conditional model. The blue and white zones collectively show values that allow persistence of the *Wolbachia* in the unconditional model, and thus, the blue zone represents *f* and *c* values where *Wolbachia* can be maintained in the unconditional model but not in the conditional model. The orange zone represents the *f* and *c* values where *Wolbachia* is eliminated in the unconditional model, but maintained in the absence of *M*

In Figure 6, we see, in the upper white zone, the relationship between *c* and *f* that permits *Wolbachia* to persist even in the presence of the conditional preference mutation, *M*. Pairs of values in this zone, such as *c* = 0.95, *f* = 0.5, allow *Wolbachia* to persist. Pairs of values below this zone, such as *c* = 0.9, *f* = 0.5 and *c* = 0.8, *f* = 0, do not allow *Wolbachia* to persist. If *c* is 1.00, then the *Wolbachia* cannot be lost in the model, as it is only *c* being less than 1.0 then causes any reduction in the *Wolbachia* frequency. But, if *c* is, for example, 0.99, and *f* = 0, Wolbachia will be lost, because the frequency of the preference allele will always increase if *f* = 0, and will eventually become so high that almost all CI will be prevented, and the *Wolbachia* will diminish in frequency by 1% per generation. But this process is slow. For example, with *f* = 0, *c* = 0.99, *x* = 1, and with initial frequencies of 0.6 for *Wolbachia* and 0.002 for the dominant preference allele, the *Wolbachia* takes 3,223 generations to drop below a 1% frequency. At this point, the frequency of the preference allele is 0.921, and only 0.6% of the population are homozygous for its absence. The blue and orange zones collectively include values of *c* and *f* where the *Wolbachia* could be stably maintained only if a conditional preference allele is not present. We note that, when the *c* and *f* values are only slightly above the grey zone, when the preference allele is present, the approach to the stable point can show cycling towards the equilibrium point, cycles that are anticlockwise if *Wolbachia* frequency is plotted on the *x*‐axis and preference mutation frequency on the *y*‐axis.

### 3.3 Simulation results: Unconditional model

Now males with the preference allele will avoid mating with females that are uninfected, whether or not the males are themselves infected. In these circumstances, the advantage for the preference allele will be less, as some full fertility crosses as well as CI crosses are being avoided by the males showing the preference.

**Figure 7:**
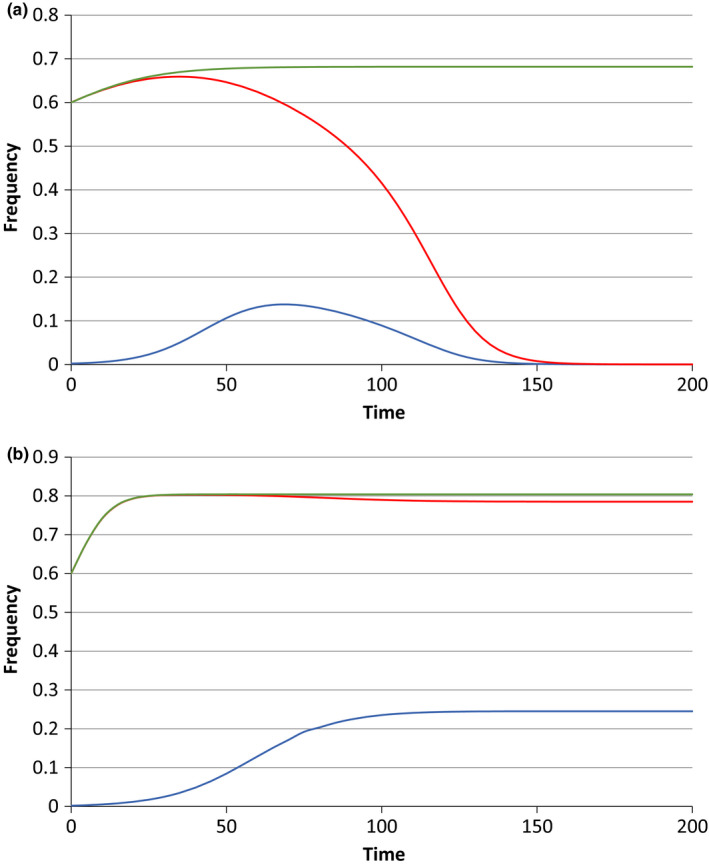
Simulations using the unconditional model, where males’ avoidance of matings with uninfected females is not conditional upon their being infected. Each figure shows the outcome when *Wolbachia* are introduced at a frequency of 0.6 and the dominant preference allele *M* at a frequency of 0.002. The upper (green) line in each case represents the *Wolbachia* frequency changes in the absence of the preference allele, the middle (red) line represents *Wolbachia* when the preference allele is introduced, and the lower (blue) line is the expected frequency changes of the preference allele. In (a), *c* is 0.88, and *f* is 0.5; the population evolves to a stable *Wolbachia* frequency of 0.682 in the absence of the preference allele, but *Wolbachia* is lost if the preference allele is introduced. (b) With *c* = 0.9 and *f* = 0.5, shows the *Wolbachia* moving to its stable frequency of 0.804 in the absence of the preference allele, which is changed to 0.785 in the presence of the preference allele, which itself evolves to a stable frequency of 0.245

Figure 7a,b show cases where the introduction of a dominant unconditional preference allele either eliminates the *Wolbachia* (7a, where *c* = 0.88, *f* = 0.5) or fails to (7b, where *c* = 0.9, *f* = 0.5). The *c* and *f* values in 7b are ones where a conditional allele can eliminate the *Wolbachia*, but the introduction of the unconditional allele, and its rise to a stable equilibrium frequency, is accompanied by a very small change in the *Wolbachia* frequency relative to its equilibrium value in the absence of the preference allele. In the case where the preference allele succeeds in eliminating the *Wolbachia*, the preference allele is itself lost, since, with a low *Wolbachia* frequency in the population, all male bearers of the preference allele are competing for the few infected females in the population and will have very few matings as a result.

As with the conditional mutation, it is possible to see what values for *c* and *f* can allow the *Wolbachia* to persist despite the presence of this mutation. Figure 6 shows, in the orange zone, the pairs of *c* and *f* values where (as in Figure 7a) the preference allele can eliminate a *Wolbachia* that is stably maintained in the absence of the preference allele, but such conditions occupy a small part of parameter space.

Strong linkage disequilibrium builds up in both the conditional and the unconditional model, that is the frequency of the *M* allele is higher in the individuals that are *I* than it is in those individuals that are *U*. In the equilibrium in Figures 3 and 4, for example, *D*′ is 0.506. In the equilibrium in Figure 7b, *D*′ is 0.746. At equilibrium, there are Hardy–Weinberg frequencies for the three genotypes *MM*, *Mm* and *mm* in *I* animals, but there is a heterozygote excess in the *U* animals. As the *M*/*m* difference is neutral in females, the frequency of *M* in *I* females will come to be the same as that in the gametes from males. At equilibrium, the *M* frequency in *I* individuals is constant, so the frequency of *M* in the male gametes that fertilize eggs from *I* mothers is the same as the *M* frequency in those eggs, and thus, with equal *M* frequencies in the two parents, the offspring will be in Hardy–Weinberg frequencies. But, in crosses involving *U* females, at equilibrium, the *M* frequency in the female gametes (which is increased each generation by the addition of formerly *I* individuals that have lost their *Wolbachia*) will be higher than in the male gametes fertilizing them, and this will give a heterozygote excess.

### 3.4 Approximate analytical results

We have looked analytically at a model where we, inaccurately, assume that there is linkage equilibrium between the presence of *Wolbachia*, represented by frequency *p*, and the *M* mutation (the symbol *r* here is used to represent the proportion of the population showing the *M* phenotype). The results from this are shown in Appendix 2. Important messages are that, for the conditional model, there is a predicted equilibrium value of *p* that is a function of *r* and of the parameters of the model, and there is a predicted value of *r* that is a function of *p* and the parameters of the model, and that, for a given parameter set, there is a pair of values of *p* and *r* that represent the joint stable equilibrium. This model with linkage equilibrium predicts a lower equilibrium frequency of the *M* mutation, and less lowering of the equilibrium *Wolbachia* frequency, than in the accurate model with linkage disequilibrium. For the unconditional model, while the analytical linkage equilibrium model predicts an equilibrium *r*, it also predicts that there is no impact of *M* on the equilibrium *Wolbachia* frequency. Thus, the impact of the preference allele on the infection in the unconditional model relies on linkage disequilibrium.

## 4 DISCUSSION

Previous work implied that there would be rapid spread of *Wolbachia* to a high stable equilibrium frequency, and the effects of this, in the potential use of *Wolbachia* to reduce the ability of wild populations of insects to act as disease vectors (Dutra et al., 2016; Hoffmann et al., 2011), has attracted great interest. Here, we see that, under simple models of male choice, there can be loss of *Wolbachia* under parameter values that would otherwise permit its stable persistence. Also, provided that the fertility of CI‐affected females is greater than zero, there can be a stable equilibrium point where both male preference and the presence of *Wolbachia* persist. Furthermore, even if the *x*, *d*, *c* and *f* values are such that this stable point exists, whether it is attained will depend on the starting frequencies of *Wolbachia* and of the preference mutation. However, in the use of *Wolbachia* to prevent disease spread, if *Wolbachia* is driven out of the population through a preference allele, it is possible that this occurs over a timescale where the loss of *Wolbachia* is slower than the loss of the disease microorganism whose transmission it prevents. If so, the impact on the targeting of the disease of the evolution of a male preference may be minor.

If a population has had its *Wolbachia* infection eliminated by a conditional preference allele, the preference allele may persist and immunize the population, to some restricted degree (see Figure 4), against subsequent *Wolbachia* invasion, although it is not clear how long such a preference allele would persist in the absence of any selection for it. A conditional preference allele has no phenotype except in the presence of *Wolbachia*. This persistence would not be seen for an unconditional preference allele, which diminishes as *Wolbachia* is eliminated. But two other outcomes of the model are identified here. One is the situation where a weak conditional preference allele can spread to 100% without eliminating the *Wolbachia*. This would result in a population where infected males showed a consistent partial avoidance of mating with uninfected females. The other situation would be the joint stable equilibrium where infected male avoidance of uninfected females could be complete or incomplete, but will be shown only by a subset of the males.

The model that has been outlined here has assumed that there is no advantage to the *Wolbachia* infection and that its spread is solely through CI. Clearly, if there was a substantial fitness gain associated with *Wolbachia*, then a preference allele that reduced or prevented CI would spread due to its selective advantage, but would not be able to eliminate the *Wolbachia* (provided that the *Wolbachia* selection exceeded [1 – *c*]). There is some evidence that *Wolbachia* can affect fitness, for example in increasing male mating rate (Champion de Crespigny, Pitt, & Wedell, 2006). But there is no consistent evidence for an effect of *Wolbachia* presence on male choosiness, for example in *Drosophila melanogaster* (Arbuthnott, Levin, & Promislow, 2016; Champion de Crespigny & Wedell, 2007). It may be that the ability of males (or females) to detect *Wolbachia* in potential partners (and in themselves) is restricted, although there is evidence that uninfected females choose uninfected males in the CI‐affected spider mite *Tetranychus urticae* (Vala, Egas, Breeuwer, & Sabelis, 2004). While an unconditional preference mutation might seem easier to achieve than a conditional mutation, it is possible that if an unconditional mutation were to spread to the joint stable equilibrium, further mutational changes that made the preference conditional upon the male infection status could spread and eliminate the *Wolbachia*. Evidence that *Wolbachia* can enhance fitness is inconsistent (Fry, Palmer, & Rand, 2004; Fry & Rand, 2002; Harcombe & Hoffmann, 2004; Ming, Shen, Cheng, Liu, & Feng, 2015). It has been argued that, since selection for alleles that prevent the effects of CI will only be strong when CI is frequent, which requires an intermediate value for the infection frequency (Sahoo, 2016), there will be few examples of evolution of host countermeasures to CI, since populations will typically be at their stable equilibria of either very high or zero infection frequencies. But our models suggest that selection for preference alleles could be strong, if mutation could produce the required alleles.

As the standard model for a *Wolbachia* that is unstable in transmission but spread by CI suggests that loss of the endosymbiont is stable, it is not clear how *Wolbachia* can ever invade, unless it conveys a direct fitness advantage in females. It could be through genetic drift. If *c* is very close to 1.00, and *f* is 0, the unstable equilibrium predicted in the absence of preference and selection is a *Wolbachia* frequency of approximately 1 − *c*, which might be attained by drift if it is just one or two per cent. An estimate of *c* in wild populations of *D. melanogaster* is 0.974 (Hoffmann et al., 1998). The population genomics of *Wolbachia* in this host shows congruence with mitochondrial DNA variants, indicating a single infection, although one that (Richardson et al., 2012) is subsequently affected by losses of *Wolbachia*, with *c* < 1.00.

We have thus seen that *Wolbachia* can potentially be eliminated from populations through the evolution of a preference allele in males that causes the avoidance of cytoplasmically incompatible crosses (just as a preference allele acting in females could also have this effect (Champion de Crespigny et al., 2005)). While a population from which *Wolbachia* has been eliminated could only show the effects of preference alleles in laboratory crosses, this study's finding that populations can (under some parameter values) evolve to situations where they can stably maintain both *Wolbachia* at intermediate frequencies and preference alleles means that wild populations could be examined to look for this combination. However, there is little current evidence that any populations with *Wolbachia* also have such preference alleles, and it may be that the challenge of having a preference phenotype that is conditional upon both an individual and its potential partners’ infection statuses may make the mutation rate to alleles with this property restrictively low.

As with any population genetics model which includes evolution towards an equilibrium state, it is uncertain whether the values of the dynamic parameters will remain constant in time for long enough for the equilibrium value defined by these parameters to be reached. The spread of *Wolbachia* through CI‐driven selection will, in most cases, be faster than its loss through incomplete transmission. However, this is a situation where the processes of sterility, preference, and incomplete transmission are intrinsic to the biology of the two interacting species, rather than being environment‐dependent. Their parameters might thus be less labile than those in models where environments have key effects.

## Appendix 1

### Equilibria generated by cytoplasmic incompatibility

*p* is the proportion of surviving offspring that come from mothers with *Wolbachia* (called *I* as opposed to *U*). These offspring are half male and half female. But the transmission of *Wolbachia* is not 100%. Rather, only a proportion c of the offspring received the *Wolbachia*. This means that the proportion of *I* zygotes, *p*′, is as follows

$$p^{\prime }=pc$$

There is then random mating, and, if an *I* male mates with a *U* female, then the fertility of the cross is *f*, where *f* < 1.

Thus, there is a loss of fitness of the population, which affects only these offspring. As a proportion *p*′ of males are *I* and a proportion (1 − *p*′) of females are *U*, and a proportion 1 − *f* of their offspring die, the population fitness is 1 − *p*′(1 − *p*′)(1 − *f*)

Thus, the frequency of *I* zygotes surviving to adulthood, *p*″, is given by

$$p\prime \prime =\frac{p\prime }{1-p\prime (1-p\prime )(1-f)}$$

But *p*′ = *pc*,

So

$$p\prime \prime =\frac{\mathrm{pc}}{1-pc(1-pc)(1-f)}$$

If we are interested in an equilibrium value of *p*, *p*″ = *p*, so

$$p\left(1-pc(1-pc)(1-f)\right)=pc$$

or

$$p\left(1-c+p^{2}c^{2}\left(1-f\right)-pc\left(1-f\right)\right)=0$$

So either *p* = 0, or

(1) $$p^{2}c^{2}\left(1-f\right)-pc\left(1-f\right)+1-c=0$$

which allows two solutions of a quadratic equation

$$p=\frac{c\left(1-f\right)\mp \sqrt{c^{2}{\left(1-f\right)}^{2}-4c^{2}\left(1-f\right)\left(1-c\right)}}{2c^{2}\left(1-f\right)}$$

which simplifies to

(2) $$p=\frac{1-f\mp \sqrt{{\left(1-f\right)}^{2}-4\left(1-f\right)\left(1-c\right)}}{2c(1-f)}$$

These solutions exist, given that the square root term is positive. This requires *f* < 4*c *− 3.

If *f* < 4*c *− 3, there is a stable equilibrium point at *p* = 0, an unstable equilibrium at

$$p=\frac{1-f-\sqrt{{\left(1-f\right)}^{2}-4\left(1-f\right)\left(1-c\right)}}{2c\left(1-f\right)}$$

and a stable equilibrium point at

(3) $$p=\frac{1-f+\sqrt{{\left(1-f\right)}^{2}-4\left(1-f\right)\left(1-c\right)}}{2c(1-f)}$$

## Appendix 2

### Approximate analytical solution for equilibria given linkage equilibrium

Here, we assume that there is linkage equilibrium between *I* and *M* alleles, and that *M* is dominant.

We consider the matings with uninfected females.

Of males available, the proportion with *M* alleles (either homozygous or heterozygous) is *r*, and the proportion infected is *pc* (since *p* is the frequency of *Wolbachia* in the mothers of the gametes making the population, and thus, *pc* is the frequency in the offspring)*.* (Note that *r* is not the frequency of the *M* allele.)

The infected males showing preference lower their matings with uninfected females by a proportion *x*. This means that the relative proportions of males of different genotypes mating with uninfected females are as follows:

Proportion of infected *M* ^+^ males = $\frac{pcr(1-x)}{1-pcrx}$
Proportion of uninfected *M* ^+^ males = $\frac{(1-pc)r}{1-pcrx}$
Proportion of infected *mm* males = $\frac{pc(1-r)}{1-pcrx}$
Proportion of uninfected *mm* males = $\frac{\left(1-pc)(1-r\right)}{1-pcrx}$

The first and third crosses have fertility *f*, and the others have fertility 1.

The *prx* infected *M* ^+^ males that have chosen not to mate with uninfected females can now compete for matings with infected females, but, as there are *pc* infected females and (1 − *pc*) uninfected females, the numbers of these males have to be multiplied by (1 − *pc*)/*pc* when they are considered as competitors for the infected females. So, for infected females, their crosses include:

Proportion of infected *M* ^+^ males = $\frac{pcr\left(1+x\frac{\left(1-pc\right)}{\mathrm{pc}}\right)}{1+pcrx\frac{1-pc}{\mathrm{pc}}}$
Proportion of uninfected *M* ^+^ males = $\frac{(1-pc)r}{1+pcrx\frac{1-pc}{\mathrm{pc}}}$
Proportion of infected *mm* males = $\frac{pc(1-r)}{1+pcrx\frac{1-pc}{\mathrm{pc}}}$
Proportion of uninfected *mm* males = $\frac{\left(1-pc)(1-r\right)}{1+pcrx\frac{1-pc}{\mathrm{pc}}}$

All these crosses have fertility 1.

Now the numbers of offspring from *M* ^+^ males and from *mm* males can be calculated, given that a proportion *pc* of crosses involve infected females and a proportion 1 − *pc* of crosses involve uninfected females, and, as the *M* ^+^ fitnesses are all a product of *r* and the *mm* are all a product of 1 − *r*, it is possible to calculate the fitnesses of *M* ^+^ and *mm* males.

$\begin{array}{cc} M^{+}\;\text{fitness}\;\text{is}\; & \frac{\left(1-pc\right)\left(pcf(1-x)+1-pc\right)}{1-pcrx}+\frac{pc\left(pc+x(1-pc)+1-pc\right)}{1+rx(1-pc)}\\ & =\frac{\left(1-pc\right)\left(pcf(1-x)+1-pc\right)}{1-pcrx}+\frac{pc\left(1+x(1-pc)\right)}{1+rx(1-pc)} \end{array}$

*mm* fitness is $\frac{\left(1-pc)(pcf+1-pc\right)}{1-pcrx}+\frac{\mathrm{pc}}{1+rx(1-pc)}$

When these fitnesses are equal,

$$\frac{\left(1-pc\right)\left(pcf\left(1-x\right)+1-pc-pcf-1+pc\right)}{1-pcrx}+\frac{pc\left(1+x\left(1-pc\right)-1\right)}{1+rx\left(1-pc\right)}=0$$

$$\frac{\left(1-pc\right)\left(-pcfx\right)}{1-pcrx}+\frac{pcx\left(1-pc\right)}{1+rx\left(1-pc\right)}=0$$

This is true if *pcx*(1 − *pc*) = 0, that is if there is no variation in the population (*pc*(1 − *pc*) = 0), or if *x* = 0 (the preference allele has no phenotype). Alternatively, it is true if

$$\frac{-f}{1-pcrx}+\frac{1}{1+rx\left(1-pc\right)}=0$$

So $\frac{-f\left(1+rx\left(1-pc\right)\right)+1-pcrx}{\left(1-pcrx\right)\left(1+rx\left(1-pc\right)\right)}=0$

The numerator thus, for equal fitness, must equal 0, and

$$-f\left(1+rx\left(1-pc\right)\right)+1-pcrx=0$$

-f-frx+frxpc+1-pcrx=0

$$1-f-r\left(fx-fxpc+pcx\right)=0$$

(4) $$r=\frac{1-f}{x\left(pc+f\left(1-pc\right)\right)}$$

This is thus the predicted equilibrium value of *r.*

Equally, for a given *r* value, we can predict the equilibrium *p*. This will come when the fitness of the population is equal to *c*.

It is *U* females that show a loss in fitness. The males that will mate with *U* females will include:

(If *x* < 1.0) *I* males with the preference mutation, which will constitute *pcr*(1 − *x*)

*I* males without the preference mutation, which will constitute *pc*(1 − *r*)

*U* males, which will constitute 1 − *pc*.

As the first two types of crosses will be CI crosses, with fertility *f*, the overall fertility (fitness) of *U* females will be

$$\frac{pcrf(1-x)+pcf(1-r)+1-pc}{pcr(1-x)+pc(1-r)+1-pc}$$

which can be simplified to

$$\frac{pcf(1-rx)+1-pc}{1-pcrx}$$

This is the fitness of *U* females, which constitute a proportion 1 − *pc* of the female population. The *pc I* females have a fitness of one. Thus, the population fitness is

$$\frac{(1-pc)\left(pcf(1-rx)+1-pc\right)+pc\left(1-pcrx\right)}{1-pcrx}$$

At equilibrium *p*, this must be equal to *c*.

Rearrangement gives $p^{2}c^{2}\left(1-f\right)\left(1-rx\right)+pc\left(f+crx-1-frx\right)+1-c=0$

There are two solutions for *p*, but the upper, stable, equilibrium is given by

(5) $$p=\frac{1+frx-f-crx+\sqrt{{\left(1+frx-f-crx\right)}^{2}-4\left(1-f\right)\left(1-c\right)\left(1-rx\right)}}{2c(1-f)(1-rx)}$$

which is the same as (3) when *r* = 0 (i.e. when there is no preference allele)

We look at our simple equilibrium when *M* is dominant, *x* = 1, *f* = 0.5 and *c* = 0.95. This moves in the simulation to a joint stable equilibrium where *p* is 0.89848, and *r* is 0.60079. The formula (4) predicts that, for *p* = .89848, equilibrium *r* should be 0.53950, and formula (5) predicts that, for *r* = 0.60079, equilibrium *p* should be 0.90403. Trial and error reveal that, using (4) and (5), the predicted joint stable equilibrium is *p* = 0.91258 and *r* = 0.53564. The positive linkage disequilibrium between the *Wolbachia* and the preference allele, which is not included in these approximate results, has the effect that, in the simulations, the preference allele achieves a higher fitness and thus frequency, since it is found preferentially in infected males, where its advantage is greater, and this linkage disequilibrium also has the effect that the preference allele is more effective in reducing the frequency of the *Wolbachia*.

Note that, when *f* = 0, (4) predicts that there can be no equilibrium *r* value that is one or less. *r* can only have a stable equilibrium value if *f* (the CI fertility) is greater than zero.

#### Unconditional model

We assume that there is linkage equilibrium between the infection and *M* allele.

We can consider the matings with uninfected females.

Of males available, the proportion with *M* alleles is *r*, and the proportion infected is *pc*.

All males showing preference lower their matings with uninfected females by a proportion *x*. This means that the relative proportions of males of different genotypes mating with uninfected females are as follows:

Proportion of infected *M* ^+^ males = $\frac{pcr(1-x)}{1-rx}$
Proportion of uninfected *M* ^+^ males = $\frac{\left(1-pc)r(1-x\right)}{1-rx}$
Proportion of infected *mm* males = $\frac{pc(1-r)}{1-rx}$
Proportion of uninfected *mm* males = $\frac{\left(1-pc)(1-r\right)}{1-rx}$

The first and third crosses have fertility *f*, and the others have fertility 1.

The *rx* infected and uninfected *M* ^+^ males that have chosen not to mate with uninfected females can now compete for matings with infected females, but, as there are *pc* infected females and (1 − *pc*) uninfected females, the numbers of these males have to be multiplied by (1 − *pc*)/*pc* when they are considered as competitors for the infected females.

Proportion of infected *M* ^+^ males = $\frac{pcr\left(1+x\frac{\left(1-pc\right)}{\mathrm{pc}}\right)}{1+rx\frac{1-pc}{\mathrm{pc}}}$
Proportion of uninfected *M* ^+^ males = $\frac{\left(1-pc\right)r\left(1+x\frac{\left(1-pc\right)}{\mathrm{pc}}\right)}{1+rx\frac{1-pc}{\mathrm{pc}}}$
Proportion of infected *mm* males = $\frac{pc(1-r)}{1+rx\frac{1-pc}{\mathrm{pc}}}$
Proportion of uninfected *mm* males = $\frac{\left(1-pc)(1-r\right)}{1+rx\frac{1-pc}{\mathrm{pc}}}$

All these crosses have fertility 1.

Now the numbers of offspring from *M* ^+^ males and from *mm* males can be calculated, given that a proportion *p*c of crosses involve infected females and a proportion 1 − *pc* of crosses involve uninfected females, and, as the *M* ^+^ fitnesses are all a product of *r* and the *mm* are all a product of 1 − *r*, it is possible to calculate the fitnesses of *M* ^+^ and *mm* males.

$\begin{array}{cc} M^{+}\;\text{fitness}\;\text{is} & \frac{(1-pc)\left(pcf(1-x)+(1-pc)(1-x)\right)}{1-rx}+\frac{pc\left(pc\left(pc+x(1-pc)\right)+(1-pc)(pc+x(1-pc)\right)}{pc+rx(1-pc)}\\ & =\frac{\left(1-pc)(1-x)(pcf+1-pc\right)}{1-rx}+\frac{pc\left(pc+x(1-pc)\right)}{pc+rx(1-pc)} \end{array}$

*mm* fitness is $\frac{\left(1-pc)(pcf+1-pc\right)}{1-pcrx}+\frac{p^{2}c^{2}}{pc+rx(1-pc)}$

When these fitnesses are equal,

$$\frac{\left(1-pc)(-x)(pcf+1-pc\right)}{1-rx}+\frac{pcx(1-pc)}{pc+rx(1-pc)}=0$$

This is true if *x*(1 − *pc*) = 0, that is all individuals are infected ((1 − *pc*) = 0), or if *x* = 0 (the preference allele has no phenotype). Alternatively, it is true if

$$\frac{pc-1-pcf}{1-rx}+\frac{\mathrm{pc}}{pc+rx(1-pc)}=0$$

So,

$$\frac{\left(pc-1-pcf\right)\left(pc+rx-rxpc\right)+pc\left(1-rx\right)}{\left(1-rx\right)\left(pc+rx(1-pc)\right)}=0$$

The numerator thus, for equal fitness, must equal 0, and

$$p^{2}c^{2}+pcrx-p^{2}c^{2}rx-pc-rx+pcrx-p^{2}c^{2}f-pcfrx+p^{2}c^{2}frx+pc-pcrx=0$$

$$p^{2}c^{2}-p^{2}c^{2}f-p^{2}c^{2}rx-rx+pcrx-pcfrx+p^{2}c^{2}frx=0$$

$$p^{2}c^{2}\left(1-f\right)-rx\left(p^{2}c^{2}+1-pc+pcf-p^{2}c^{2}f\right)=0$$

$$p^{2}c^{2}\left(1-f\right)-rx\left(1-pc(1-f)(1-pc)\right)=0$$

(6) $$r=\frac{p^{2}c^{2}\left(1-f\right)}{x\left(1-pc\left(1-f\right)\left(1-pc\right)\right)}$$

This is thus the predicted equilibrium value of *r*.

Simulations of an unconditional dominant preference allele with *x* = 1, *f* = 0.5 and *c* = 0.90 gives a joint stable equilibrium where *p* is 0.785056 and *r* is 0.422125 (summing homozygotes and heterozygotes for *M*). The simulated value of* r* is very much greater than the value of 0.27848 predicted by (6).

What is the equilibrium *p*? This will come when the fitness of the population is equal to *c*.

It is *U* females that show a loss in fitness. The males that will mate with *U* females will include:

(If *x* < 1.0) *I* males with the preference mutation, which will constitute *pcr*(1 − *x*)

*I* males without the preference mutation, which will constitute *pc*(1 − *r*)

(If *x* < 1.0) *U* males with the preference mutation, which will constitute (1 − *pc*)*r*(1 − *x*)

*U* males without the preference mutation, which will constitute (1 − *pc*)(1 − *r*)

As the first two types of crosses will be CI crosses, with fertility *f*, the overall fertility (fitness) of *U* females will be

$$\frac{pcrf\left(1-x\right)+pcf\left(1-r\right)+\left(1-pc\right)\left(r-rx+1-r\right)}{pcr\left(1-x\right)+pc\left(1-r\right)+\left(1-pc\right)\left(r-rx+1-r\right)}$$

which can be simplified to

$$\frac{\left(pcf+1-pc\right)\left(1-rx\right)}{1-rx}$$

or pcf+1-pc.

This is the fitness of *U* females, which constitute a proportion 1 − *pc* of the female population. The *pc I* females have a fitness of one. Thus, the population fitness is

(1-pc)(pcf+1-pc)+pc.

At equilibrium *p*, this must be equal to *c*.

Rearrangement gives $p^{2}c^{2}+pc\left(f-1\right)+1-c=0,$ which is the same as (1). So, in the unconditional model, there is no predicted effect of the preference allele on the *Wolbachia* infection. However, our simulations show that the preference allele has an effect, and, indeed, can drive the *Wolbachia* infection extinct. But this is entirely due to the effect of linkage disequilibrium, where the preference allele lowers the frequency of *Wolbachia* because it is associated with the presence of *Wolbachia* in males and thus reduces CI crosses between *I* males and *U* females more than it reduces the non‐CI crosses between *U* males and *U* females.

## References

[jeb13602-bib-0001] Arbuthnott, D. , Levin, T. C. , & Promislow, D. E. L. (2016). The impacts of *Wolbachia* and the microbiome on mate choice in *Drosophila melanogaster* . Journal of Evolutionary Biology, 29, 461–468. 26548557 10.1111/jeb.12788 PMC4744502

[jeb13602-bib-0002] Caspari, E. , & Watson, G. S. (1959). On the evolutionary importance of cytoplasmic sterility in mosquitoes. Evolution, 13, 568–570. 10.1111/j.1558-5646.1959.tb03045.x

[jeb13602-bib-0003] Champion de Crespigny, F. E. , Butlin, R. K. , & Wedell, N. (2005). Can cytoplasmic incompatibility inducing *Wolbachia* promote the evolution of mate preferences? Journal of Evolutionary Biology, 18, 967–977. 10.1111/j.1420-9101.2005.00909.x 16033569

[jeb13602-bib-0004] Champion de Crespigny, F. E. , Pitt, T. D. , & Wedell, N. (2006). Increased male mating rate in *Drosophila* is associated with Wolbachia infection. Journal of Evolutionary Biology, 19, 1964–1972. 10.1111/j.1420-9101.2006.01143.x 17040394

[jeb13602-bib-0005] Champion de Crespigny, F. E. , & Wedell, N. (2007). Mate preferences in *Drosophila* infected with Wolbachia? Behavioral Ecology and Sociobiology, 61, 1229–1235.

[jeb13602-bib-0006] Dutra, H. L. C. , Rocha, M. N. , Dias, F. B. S. , Mansur, S. B. , Caragata, E. P. , & Moreira, L. A. (2016). *Wolbachia* blocks currently circulating zika virus isolates in Brazilian *Aedes aegypti* mosquitoes. Cell Host and Microbe, 19, 771–774. 10.1016/j.chom.2016.04.021 27156023 PMC4906366

[jeb13602-bib-0007] Engelstädter, J. , & Hurst, G. D. D. (2009). The ecology and evolution of microbes that manipulate host reproduction. Annual Review of Ecology, Evolution, and Systematics, 40, 127–149. 10.1146/annurev.ecolsys.110308.120206

[jeb13602-bib-0008] Fry, A. J. , Palmer, M. R. , & Rand, D. M. (2004). Variable fitness effects of Wolbachia infection in *Drosophila melanogaster* . Heredity, 93, 379–389. 10.1038/sj.hdy.6800514 15305172

[jeb13602-bib-0009] Fry, A. J. , & Rand, D. M. (2002). Wolbachia interactions that determine *Drosophila melanogaster* survival. Evolution, 56, 1976–1981. 10.1111/j.0014-3820.2002.tb00123.x 12449484

[jeb13602-bib-0010] Harcombe, W. , & Hoffmann, A. A. (2004). Wolbachia effects in *Drosophila melanogaster*: In search of fitness benefits. Journal of Invertebrate Pathology, 87, 45–50. 10.1016/j.jip.2004.07.003 15491598

[jeb13602-bib-0011] Hoffmann, A. A. , Hercus, M. , & Dagher, H. (1998). Population dynamics of the Wolbachia infection causing cytoplasmic incompatibility in *Drosophila melanogaster* . Genetics, 148, 221–231. 9475734 10.1093/genetics/148.1.221 PMC1459765

[jeb13602-bib-0012] Hoffmann, A. A. , Montgomery, B. L. , Popovici, J. , Iturbe‐Ormaetxe, I. , Johnson, P. H. , Muzzi, F. , … O’Neill, S. L. (2011). Successful establishment of *Wolbachia* in *Aedes* populations to suppress dengue transmission. Nature, 476, 454–459. 10.1038/nature10356 21866160

[jeb13602-bib-0013] Ming, Q.‐L. , Shen, J.‐F. , Cheng, C. , Liu, C.‐M. , & Feng, Z.‐J. (2015). *Wolbachia* infection dynamics in *Tribolium confusum* (Coleoptera: Tenebrionidae) and their effects on host mating behaviour and reproduction. Journal of Economic Entomology, 108, 1408–1415. 26470269 10.1093/jee/tov053

[jeb13602-bib-0014] Moreira, L. A. , Iturbe‐Ormaetxe, I. , Jeffery, J. A. , Lu, G. , Pyke, A. T. , Hedges, L. M. , … O’Neill, S. L. (2009). A *Wolbachia* symbiont in *Aedes aegypti* limits infection with dengue, chikungunya and *Plasmodium* . Cell, 139, 1268–1278. 10.1016/j.cell.2009.11.042 20064373

[jeb13602-bib-0015] Richardson, M. F. , Weinert, L. A. , Welch, J. J. , Linheiro, R. S. , Magwire, M. M. , Jiggins, F. M. , & Bergman, C. M. (2012). Population genomics of the *Wolbachia* endosymbiont in *Drosophila melanogaster* . PLoS Genetics, 8, e1003129 10.1371/journal.pgen.1003129 23284297 PMC3527207

[jeb13602-bib-0016] Sahoo, R. K. (2016). Why antagonistic traits against cytoplasmic incompatibility are so elusive. Frontiers in Microbiology, 7, 392 10.3389/fmicb.2016.00392 27065360 PMC4814451

[jeb13602-bib-0017] Turelli, M. , & Hoffmann, A. A. (1991). Rapid spread of an inherited incompatibility factor in California *Drosophila* . Nature, 353, 440–442. 10.1038/353440a0 1896086

[jeb13602-bib-0018] Turelli, M. , & Hoffmann, A. A. (1995). Cytoplasmic incompatibility in *Drosophila simulans*: Dynamics and parameter estimates from natural populations. Genetics, 140, 1319–1338. 7498773 10.1093/genetics/140.4.1319 PMC1206697

[jeb13602-bib-0019] Vala, F. , Egas, M. , Breeuwer, J. A. J. , & Sabelis, M. W. (2004). Wolbachia affects oviposition and mating behaviour of its spider mite host. Journal of Evolutionary Biology, 17, 692–700. 10.1046/j.1420-9101.2003.00679.x 15149411

[jeb13602-bib-0020] Werren, J. H. , Baldo, L. , & Clark, M. E. (2008). *Wolbachia*: Master manipulators of invertebrate biology. Nature Reviews Microbiology, 6, 741–751. 10.1038/nrmicro1969 18794912

