# Supplementary material for: Alternative evolutionary outcomes following endosymbiont‐mediated selection on male mating preference alleles
Source: J Evol Biol. 2020 Feb 21;33(5):653–67. doi: 10.1111/jeb.13602 (PMC7318244; doi:10.1111/jeb.13602)
Supplement: Supplementary file 1 [file JEB-33-653-s001.docx]

**Electronic Supplementary Material**

**Conditional Program**

#

#include <cstdio>

#include <math.h>

#include <cstdlib>

#include <iostream>

using namespace std;

int main(int nNumberofArgs, char* pszArgs[])

{

float c,r, LD1,LD2,LD3,LD4, meanfitness,f,p,pM, denom1, denom;

float WMM,WMm, Wmm, wMM, wMm, wmm, W, M, x, d, D,Dprime, LDmax, LDmax1, LDmax2;

float WMM1,WMm1, Wmm1, wMM1, wMm1, wmm1,pW,pw,Whet, whet;

float WMM2,WMm2, Wmm2, wMM2, wMm2, wmm2;

int i, generations;

cout << "What is transmission rate?";

cin >> c;

cout << "What is CI fertility?";

cin >> f;

cout << "What is the initial frequency of Wolbachia?";

cin >> p;

cout << "What is the initial frequency of the choice mutation?";

cin >> pM;

cout << "How strong is the effect of the choice mutation?";

cin >> x;

cout << "What is dominance?";

cin >> d;

cout << "How many generations?";

cin >> generations;

//set up initial frequncies of the six genotypes, assuming Hardy-Weinberg and LD

WMM=p*pM*pM;

WMm=2*p*pM*(1-pM);

Wmm=p*(1-pM)*(1-pM);

wMM=(1-p)*pM*pM;

wMm=2*(1-p)*pM*(1-pM);

wmm=(1-p)*(1-pM)*(1-pM);

// Go through generations of change of the six genotypes

for (i=1; i<generations +1; i++)

{

//Include the effects of incomplete transmission (c<1)

WMM1=WMM*c;

WMm1=WMm*c;

Wmm1=Wmm*c;

wMM1=wMM+WMM*(1-c);

wMm1=wMm+WMm*(1-c);

wmm1=wmm+Wmm*(1-c);

//Set the six genotypes that will constitute the offspring to zero

WMM2=0;

WMm2=0;

Wmm2=0;

wMM2=0;

wMm2=0;

wmm2=0;

meanfitness=0;

//Calculate total male crosses with uninfected females, given the preference

denom=1-WMM1*x-WMm1*x*d;

p=WMM1+WMm1+Wmm1;

if (p<0.0001)

p=0.0001;

//p is adjusted to prevent division by zero;

//calculate total male crosses with infected females, given the preference

denom1=1+(WMM1*x+WMm1*x*d)*(1-p)/p;

//Work through crosses - homozygous infected preference females with different males

WMM2+=WMM1*WMM1*(p+(1-p)*x)/(p*denom1);

meanfitness+=WMM1*WMM1*(p+(1-p)*x)/(p*denom1);

WMM2+=0.5*WMM1*WMm1*(p+(1-p)*x*d)/(p*denom1);

WMm2+=0.5*WMM1*WMm1*(p+(1-p)*x*d)/(p*denom1);

meanfitness+=WMM1*WMm1*(p+(1-p)*x*d)/(p*denom1);

WMm2+=WMM1*Wmm1/denom1;

meanfitness+=WMM1*Wmm1/denom1;

WMM2+=WMM1*wMM1/denom1;

meanfitness+=WMM1*wMM1/denom1;

WMM2+=0.5*WMM1*wMm1/denom1;

WMm2+=0.5*WMM1*wMm1/denom1;

meanfitness+=WMM1*wMm1/denom1;

WMm2+=WMM1*wmm1/denom1;

meanfitness+=WMM1*wmm1/denom1;

//Now infected heterozygous preference females with various males

WMM2+=0.5*WMm1*WMM1*(p+(1-p)*x)/(p*denom1);

WMm2+=0.5*WMm1*WMM1*(p+(1-p)*x)/(p*denom1);

meanfitness+=WMm1*WMM1*(p+(1-p)*x)/(p*denom1);

WMM2+=0.25*WMm1*WMm1*(p+(1-p)*x*d)/(p*denom1);

WMm2+=0.5*WMm1*WMm1*(p+(1-p)*x*d)/(p*denom1);

Wmm2+=0.25*WMm1*WMm1*(p+(1-p)*x*d)/(p*denom1);

meanfitness+=WMm1*WMm1*(p+(1-p)*x*d)/(p*denom1);

WMm2+=0.5*WMm1*Wmm1/denom1;

Wmm2+=0.5*WMm1*Wmm1/denom1;

meanfitness+=WMm1*Wmm1/denom1;

WMM2+=0.5*WMm1*wMM1/denom1;

WMm2+=0.5*WMm1*wMM1/denom1;

meanfitness+=WMm1*wMM1/denom1;

WMM2+=0.25*WMm1*wMm1/denom1;

WMm2+=0.5*WMm1*wMm1/denom1;

Wmm2+=0.25*WMm1*wMm1/denom1;

meanfitness+=WMm1*wMm1/denom1;

WMm2+=0.5*WMm1*wmm1/denom1;

Wmm2+=0.5*WMm1*wmm1/denom1;

meanfitness+=WMm1*wmm1/denom1;

//Now infected homozygous non-preference females with various males

WMm2+=Wmm1*WMM1*(p+(1-p)*x)/(p*denom1);

meanfitness+=Wmm1*WMM1*(p+(1-p)*x)/(p*denom1);

WMm2+=0.5*Wmm1*WMm1*(p+(1-p)*x*d)/(p*denom1);

Wmm2+=0.5*Wmm1*WMm1*(p+(1-p)*x*d)/(p*denom1);

meanfitness+=Wmm1*WMm1*(p+(1-p)*x*d)/(p*denom1);

Wmm2+=Wmm1*Wmm1/denom1;

meanfitness+=Wmm1*Wmm1/denom1;

WMm2+=Wmm1*wMM1/denom1;

meanfitness+=Wmm1*wMM1/denom1;

WMm2+=0.5*Wmm1*wMm1/denom1;

Wmm2+=0.5*Wmm1*wMm1/denom1;

meanfitness+=Wmm1*wMm1/denom1;

Wmm2+=Wmm1*wmm1/denom1;

meanfitness+=Wmm1*wmm1/denom1;

//Uninfected homozygous preference females with various males

wMM2+=wMM1*WMM1*(1-x)*f/denom;

meanfitness+=wMM1*WMM1*(1-x)*f/denom;

wMM2+=0.5*wMM1*WMm1*(1-x*d)*f/denom;

wMm2+=0.5*wMM1*WMm1*(1-x*d)*f/denom;

meanfitness+=wMM1*WMm1*(1-x*d)*f/denom;

wMm2+=wMM1*Wmm1*f/denom;

meanfitness+=wMM1*Wmm1*f/denom;

wMM2+=wMM1*wMM1/denom;

meanfitness+=wMM1*wMM1/denom;

wMM2+=0.5*wMM1*wMm1/denom;

wMm2+=0.5*wMM1*wMm1/denom;

meanfitness+=wMM1*wMm1/denom;

wMm2+=wMM1*wmm1/denom;

meanfitness+=wMM1*wmm1/denom;

//Uninfected heterozygous preference females with various males

wMM2+=0.5*wMm1*WMM1*(1-x)*f/denom;

wMm2+=0.5*wMm1*WMM1*(1-x)*f/denom;

meanfitness+=wMm1*WMM1*(1-x)*f/denom;

wMM2+=0.25*wMm1*WMm1*(1-x*d)*f/denom;

wMm2+=0.5*wMm1*WMm1*(1-x*d)*f/denom;

wmm2+=0.25*wMm1*WMm1*(1-x*d)*f/denom;

meanfitness+=wMm1*WMm1*(1-x*d)*f/denom;

wMm2+=0.5*wMm1*Wmm1*f/denom;

wmm2+=0.5*wMm1*Wmm1*f/denom;

meanfitness+=wMm1*Wmm1*f/denom;

wMM2+=0.5*wMm1*wMM1/denom;

wMm2+=0.5*wMm1*wMM1/denom;

meanfitness+=wMm1*wMM1/denom;

wMM2+=0.25*wMm1*wMm1/denom;

wMm2+=0.5*wMm1*wMm1/denom;

wmm2+=0.25*wMm1*wMm1/denom;

meanfitness+=wMm1*wMm1/denom;

wMm2+=0.5*wMm1*wmm1/denom;

wmm2+=0.5*wMm1*wmm1/denom;

meanfitness+=wMm1*wmm1/denom;

//Uninfected homozygous non-preference females with various males

wMm2+=wmm1*WMM1*(1-x)*f/denom;

meanfitness+=wmm1*WMM1*(1-x)*f/denom;

wMm2+=0.5*wmm1*WMm1*(1-x*d)*f/denom;

wmm2+=0.5*wmm1*WMm1*(1-x*d)*f/denom;

meanfitness+=wmm1*WMm1*(1-x*d)*f/denom;

wmm2+=wmm1*Wmm1*f/denom;

meanfitness+=wmm1*Wmm1*f/denom;

wMm2+=wmm1*wMM1/denom;

meanfitness+=wmm1*wMM1/denom;

wMm2+=0.5*wmm1*wMm1/denom;

wmm2+=0.5*wmm1*wMm1/denom;

meanfitness+=wmm1*wMm1/denom;

wmm2+=wmm1*wmm1/denom;

meanfitness+=wmm1*wmm1/denom;

//Adjust all six genotype frequencies using the population's mean fitness,

//so these sum to one.

WMM=WMM2/meanfitness;

WMm=WMm2/meanfitness;

Wmm=Wmm2/meanfitness;

wMM=wMM2/meanfitness;

wMm=wMm2/meanfitness;

wmm=wmm2/meanfitness;

//Calculate proportions with Wolbachia, frequency of M and proportion with M

W=WMM+WMm+Wmm;

M=WMM+WMm/2+wMM+wMm/2;

r=WMM+WMm+wMM+wMm;

//Calculate M frequency in infected and uninfected animals

pW=(WMM+WMm/2)/W;

pw=(wMM+wMm/2)/(1-W);

//Calculate expected heterozygotes (from H-W)in infected and uninfecteds

Whet=2*pW*(1-pW)*W;

whet=2*pw*(1-pw)*(1-W);

//Print out results each generation

cout << "generation: ";

cout << i;

cout << " wol f is ";

cout << W;

cout << " Mut f is ";

cout << M;

cout << "r is ";

cout << r;

cout << "fitness is ";

cout << meanfitness<< endl;

cout << "WMM:";

cout << WMM;

cout << "WMm: ";

cout << WMm;

cout << "Wmm: ";

cout << Wmm;

cout << "wMM: ";

cout << wMM;

cout << "wMm: ";

cout << wMm;

cout << "wmm: ";

cout << wmm <<endl;

cout << "Expected hets with W = ";

cout << Whet;

cout << " Expected hets with w = ";

cout << whet << endl;

//Linkage disequilibrium- the four gamete types

LD1=WMM+WMm/2;

LD2=Wmm+WMm/2;

LD3=wMM+wMm/2;

LD4=wmm+wMm/2;

D=LD1*LD4-LD2*LD3;

LDmax1=W*(1-M);

LDmax2=(1-W)*M;

if (LDmax1<=LDmax2)

LDmax=LDmax1;

if (LDmax2<LDmax1)

LDmax=LDmax2;

Dprime=D/LDmax;

cout << "LD is D= ";

cout << D;

cout << " and D' = ";

cout << Dprime << endl;

}

//This is the end of the loop for generations

system ("PAUSE");

return 0;

}

**Unconditional Program**

#

#include <cstdio>

#include <math.h>

#include <cstdlib>

#include <iostream>

using namespace std;

int main(int nNumberofArgs, char* pszArgs[])

{

float c, r, LD1,LD2,LD3,LD4, meanfitness,f,p,pM, denom1, denom;

float WMM,WMm, Wmm, wMM, wMm, wmm, W, M, x, d, D,Dprime, LDmax, LDmax1, LDmax2;

float WMM1,WMm1, Wmm1, wMM1, wMm1, wmm1,Whet, whet, pw, pW;

float WMM2,WMm2, Wmm2, wMM2, wMm2, wmm2;

int i, generations;

cout << "What is transmission rate?";

cin >> c;

cout << "What is CI fertility?";

cin >> f;

cout << "What is the initial frequency of Wolbachia?";

cin >> p;

cout << "What is the initial frequency of the choice mutation?";

cin >> pM;

cout << "How strong is the effect of the choice mutation?";

cin >> x;

cout << "What is dominance?";

cin >> d;

cout << "How many generations?";

cin >> generations;

//Set up initial frequncies of the six genotypes, assuming Hardy-Weinberg and LD

WMM=p*pM*pM;

WMm=2*p*pM*(1-pM);

Wmm=p*(1-pM)*(1-pM);

wMM=(1-p)*pM*pM;

wMm=2*(1-p)*pM*(1-pM);

wmm=(1-p)*(1-pM)*(1-pM);

//Go through generations of change of the six genotypes

for (i=1; i<generations +1; i++)

{

//Include the effects of incomplete transmission (c<1)

WMM1=WMM*c;

WMm1=WMm*c;

Wmm1=Wmm*c;

wMM1=wMM+WMM*(1-c);

wMm1=wMm+WMm*(1-c);

wmm1=wmm+Wmm*(1-c);

//Set the six genotypes that will constitute the offspring to zero

WMM2=0;

WMm2=0;

Wmm2=0;

wMM2=0;

wMm2=0;

wmm2=0;

meanfitness=0;

//Calculate total male crosses with uninfected females, given the preference

denom=1-WMM1*x-WMm1*x*d-wMM1*x-wMm*x*d;

p=WMM1+WMm1+Wmm1;

if (p<0.0001)

p=0.0001;

//p is adjusted to prevent division by zero.

//Calculate total male crosses with infected females, given the preference

denom1=1+(WMM1*x+WMm1*x*d+wMM1*x+wMm1*x*d)*(1-p)/p;

//Work through crosses, infected homozygous preference females with various males

WMM2+=WMM1*WMM1*(p+(1-p)*x)/(p*denom1);

meanfitness+=WMM1*WMM1*(p+(1-p)*x)/(p*denom1);

WMM2+=0.5*WMM1*WMm1*(p+(1-p)*x*d)/(p*denom1);

WMm2+=0.5*WMM1*WMm1*(p+(1-p)*x*d)/(p*denom1);

meanfitness+=WMM1*WMm1*(p+(1-p)*x*d)/(p*denom1);

WMm2+=WMM1*Wmm1/denom1;

meanfitness+=WMM1*Wmm1/denom1;

WMM2+=WMM1*wMM1*(p+(1-p)*x)/(p*denom1);

meanfitness+=WMM1*wMM1*(p+(1-p)*x)/(p*denom1);

WMM2+=0.5*WMM1*wMm1*(p+(1-p)*x*d)/(p*denom1);

WMm2+=0.5*WMM1*wMm1*(p+(1-p)*x*d)/(p*denom1);

meanfitness+=WMM1*wMm1*(p+(1-p)*x*d)/(p*denom1);

WMm2+=WMM1*wmm1/denom1;

meanfitness+=WMM1*wmm1/denom1;

//Infected heterozygous preference females with various males

WMM2+=0.5*WMm1*WMM1*(p+(1-p)*x)/(p*denom1);

WMm2+=0.5*WMm1*WMM1*(p+(1-p)*x)/(p*denom1);

meanfitness+=WMm1*WMM1*(p+(1-p)*x)/(p*denom1);

WMM2+=0.25*WMm1*WMm1*(p+(1-p)*x*d)/(p*denom1);

WMm2+=0.5*WMm1*WMm1*(p+(1-p)*x*d)/(p*denom1);

Wmm2+=0.25*WMm1*WMm1*(p+(1-p)*x*d)/(p*denom1);

meanfitness+=WMm1*WMm1*(p+(1-p)*x*d)/(p*denom1);

WMm2+=0.5*WMm1*Wmm1/denom1;

Wmm2+=0.5*WMm1*Wmm1/denom1;

meanfitness+=WMm1*Wmm1/denom1;

WMM2+=0.5*WMm1*wMM1*(p+(1-p)*x)/(p*denom1);

WMm2+=0.5*WMm1*wMM1*(p+(1-p)*x)/(p*denom1);

meanfitness+=WMm1*wMM1*(p+(1-p)*x)/(p*denom1);

WMM2+=0.25*WMm1*wMm1*(p+(1-p)*x*d)/(p*denom1);

WMm2+=0.5*WMm1*wMm1* (p+(1-p)*x*d)/(p*denom1);

Wmm2+=0.25*WMm1*wMm1*(p+(1-p)*x*d)/(p*denom1);

meanfitness+=WMm1*wMm1*(p+(1-p)*x*d)/(p*denom1);

WMm2+=0.5*WMm1*wmm1/denom1;

Wmm2+=0.5*WMm1*wmm1/denom1;

meanfitness+=WMm1*wmm1/denom1;

//Infected homozygous non-preference females with various males

WMm2+=Wmm1*WMM1*(p+(1-p)*x)/(p*denom1);

meanfitness+=Wmm1*WMM1*(p+(1-p)*x)/(p*denom1);

WMm2+=0.5*Wmm1*WMm1*(p+(1-p)*x*d)/(p*denom1);

Wmm2+=0.5*Wmm1*WMm1*(p+(1-p)*x*d)/(p*denom1);

meanfitness+=Wmm1*WMm1*(p+(1-p)*x*d)/(p*denom1);

Wmm2+=Wmm1*Wmm1/denom1;

meanfitness+=Wmm1*Wmm1/denom1;

WMm2+=Wmm1*wMM1*(p+(1-p)*x)/(p*denom1);

meanfitness+=Wmm1*wMM1*(p+(1-p)*x)/(p*denom1);

WMm2+=0.5*Wmm1*wMm1*(p+(1-p)*x*d)/(p*denom1);

Wmm2+=0.5*Wmm1*wMm1*(p+(1-p)*x*d)/(p*denom1);

meanfitness+=Wmm1*wMm1*(p+(1-p)*x*d)/(p*denom1);

Wmm2+=Wmm1*wmm1/denom1;

meanfitness+=Wmm1*wmm1/denom1;

//Uninfected homozygous preference females with various males

wMM2+=wMM1*WMM1*(1-x)*f/denom;

meanfitness+=wMM1*WMM1*(1-x)*f/denom;

wMM2+=0.5*wMM1*WMm1*(1-x*d)*f/denom;

wMm2+=0.5*wMM1*WMm1*(1-x*d)*f/denom;

meanfitness+=wMM1*WMm1*(1-x*d)*f/denom;

wMm2+=wMM1*Wmm1*f/denom;

meanfitness+=wMM1*Wmm1*f/denom;

wMM2+=wMM1*wMM1*(1-x)/denom;

meanfitness+=wMM1*wMM1*(1-x)/denom;

wMM2+=0.5*wMM1*wMm1*(1-x*d)/denom;

wMm2+=0.5*wMM1*wMm1*(1-x*d)/denom;

meanfitness+=wMM1*wMm1*(1-x*d)/denom;

wMm2+=wMM1*wmm1/denom;

meanfitness+=wMM1*wmm1/denom;

//Uninfected heterozygous preference females with various males

wMM2+=0.5*wMm1*WMM1*(1-x)*f/denom;

wMm2+=0.5*wMm1*WMM1*(1-x)*f/denom;

meanfitness+=wMm1*WMM1*(1-x)*f/denom;

wMM2+=0.25*wMm1*WMm1*(1-x*d)*f/denom;

wMm2+=0.5*wMm1*WMm1*(1-x*d)*f/denom;

wmm2+=0.25*wMm1*WMm1*(1-x*d)*f/denom;

meanfitness+=wMm1*WMm1*(1-x*d)*f/denom;

wMm2+=0.5*wMm1*Wmm1*f/denom;

wmm2+=0.5*wMm1*Wmm1*f/denom;

meanfitness+=wMm1*Wmm1*f/denom;

wMM2+=0.5*wMm1*wMM1*(1-x)/denom;

wMm2+=0.5*wMm1*wMM1*(1-x)/denom;

meanfitness+=wMm1*wMM1*(1-x)/denom;

wMM2+=0.25*wMm1*wMm1*(1-x*d)/denom;

wMm2+=0.5*wMm1*wMm1*(1-x*d)/denom;

wmm2+=0.25*wMm1*wMm1*(1-x*d)/denom;

meanfitness+=wMm1*wMm1*(1-x*d)/denom;

wMm2+=0.5*wMm1*wmm1/denom;

wmm2+=0.5*wMm1*wmm1/denom;

meanfitness+=wMm1*wmm1/denom;

//Uninfected homozygous non-preference females with various males

wMm2+=wmm1*WMM1*(1-x)*f/denom;

meanfitness+=wmm1*WMM1*(1-x)*f/denom;

wMm2+=0.5*wmm1*WMm1*(1-x*d)*f/denom;

wmm2+=0.5*wmm1*WMm1*(1-x*d)*f/denom;

meanfitness+=wmm1*WMm1*(1-x*d)*f/denom;

wmm2+=wmm1*Wmm1*f/denom;

meanfitness+=wmm1*Wmm1*f/denom;

wMm2+=wmm1*wMM1*(1-x)/denom;

meanfitness+=wmm1*wMM1*(1-x)/denom;

wMm2+=0.5*wmm1*wMm1*(1-x*d)/denom;

wmm2+=0.5*wmm1*wMm1*(1-x*d)/denom;

meanfitness+=wmm1*wMm1*(1-x*d)/denom;

wmm2+=wmm1*wmm1/denom;

meanfitness+=wmm1*wmm1/denom;

//cout << "The values are now, after wmm ";

//Adjust all six genotype frequencies using he mean fitness,

//so that these sum to one

WMM=WMM2/meanfitness;

WMm=WMm2/meanfitness;

Wmm=Wmm2/meanfitness;

wMM=wMM2/meanfitness;

wMm=wMm2/meanfitness;

wmm=wmm2/meanfitness;

//\\\calculate proportions with Wolbachia, frequency of M and proportion with M

W=WMM+WMm+Wmm;

M=WMM+WMm/2+wMM+wMm/2;

r=WMM+WMm+wMM+wMm;

//Calculate M frequency in infected and uninfected animals

pW=(WMM+WMm/2)/W;

pw=(wMM+wMm/2)/(1-W);

//Calculate expected heterozygotes (from H-W)in infected and uninfecteds

Whet=2*pW*(1-pW)*W;

whet=2*pw*(1-pw)*(1-W);

//Print out results each generation

cout << "generation: ";

cout << i;

cout << " wol f is ";

cout << W;

cout << " Mut f is ";

cout << M;

cout << " r is ";

cout << r;

cout << "fitness is ";

cout << meanfitness<< endl;

cout << "Classes are: WMM ";

cout << WMM;

cout << " WMm: ";

cout << WMm;

cout << " Wmm: ";

cout << Wmm;

cout << " wMM: ";

cout << wMM;

cout << " wMm: ";

cout << wMm;

cout << " wmm: ";

cout << wmm << endl;

cout << "Expected hets with W = ";

cout << Whet;

cout << " Expected hets with w = ";

cout << whet << endl;

//Linkage disequilibrium - the four gamete types

LD1=WMM+WMm/2;

LD2=Wmm+WMm/2;

LD3=wMM+wMm/2;

LD4=wmm+wMm/2;

D=(LD1*LD4)-(LD2*LD3);

LDmax1=W*(1-M);

LDmax2=(1-W)*M;

if (LDmax1<=LDmax2)

LDmax=LDmax1;

if (LDmax2<LDmax1)

LDmax=LDmax2;

Dprime=D/LDmax;

cout << "LD is D = ";

cout << D;

cout << " and D' = ";

cout << Dprime << endl;

}

//This is the end of the loop for generations

system ("PAUSE");

return 0;

}
